# Dihydroorotate dehydrogenase inhibition reveals metabolic vulnerability in chronic myeloid leukemia

**DOI:** 10.1038/s41419-022-05028-9

**Published:** 2022-06-30

**Authors:** Mohammad Houshmand, Nicoletta Vitale, Francesca Orso, Alessandro Cignetti, Ivan Molineris, Valentina Gaidano, Stefano Sainas, Marta Giorgis, Donatella Boschi, Carmen Fava, Alice Passoni, Marta Gai, Massimo Geuna, Federica Sora, Alessandra Iurlo, Elisabetta Abruzzese, Massimo Breccia, Olga Mulas, Giovanni Caocci, Fausto Castagnetti, Daniela Taverna, Salvatore Oliviero, Fabrizio Pane, Marco Lucio Lolli, Paola Circosta, Giuseppe Saglio

**Affiliations:** 1 Department of Clinical and Biological Sciences, University of Turin, Turin, Italy; 2 Department of Medical Sciences, University of Turin, Turin, Italy; 3 Department of Molecular Biotechnology and Health Sciences, University of Turin, Turin, Italy; 4 Department of Hematology and Cell Therapy, A.O. Ordine Mauriziano, Turin, Italy; 5 Department of Life Sciences and Systems Biology, University of Turin, Turin, Italy; 6 IIGM - Italian Institute for Genomic Medicine, c/o IRCCS, Candiolo, Italy; 7 Division of Hematology, A.O. SS Antonio e Biagio e Cesare Arrigo, Alessandria, Italy; 8 Department of Drug Science and Technology, University of Turin, Turin, Italy; 9 Mass spectrometry Laboratory, Environmental Health Sciences Department, Institute for Pharmacological Research Mario Negri Institute IRCCS, Milan, Italy; 10 Laboratory of Immunopathology, Division of Pathology, A.O. Ordine Mauriziano, Turin, Italy; 11 Fondazione Policlinico Universitario A Gemelli-IRCCS, Istituto di Ematologia Università Cattolica Sacro Cuore, Rome, Italy; 12 Hematology Division, Foundation Istituto di Ricovero e Cura a Carattere Scientifico (IRCCS) Ca’ Granda Ospedale Maggiore Policlinico, University of Milan, Milan, Italy; 13 Hematology Unit, S. Eugenio Hospital, ASLRoma2, Rome, Italy; 14 Division of Hematology, Department of Cellular Biotechnologies and Hematology, Sapienza University, Rome, Italy; 15 Department of Medical Sciences and Public Health, University of Cagliari, Businco Hospital, Cagliari, Italy; 16 Institute of Hematology “L. and A. Seràgnoli”, Department of Experimental, Diagnostic and Specialty Medicine, University of Bologna, “S. Orsola-Malpighi” Hospital, Bologna, Italy; 17 Department of Clinical Medicine and Surgery, Hematology Section, University of Naples “Federico II”, Naples, Italy

**Keywords:** Cancer metabolism, Apoptosis

## Abstract

The development of different generations of BCR-ABL1 tyrosine kinase inhibitors (TKIs) has led to the high overall survival of chronic myeloid leukemia (CML) patients. However, there are CML patients who show resistance to TKI therapy and are prone to progress to more advanced phases of the disease. So, implementing an alternative approach for targeting TKIs insensitive cells would be of the essence. Dihydroorotate dehydrogenase (DHODH) is an enzyme in the de novo pyrimidine biosynthesis pathway that is located in the inner membrane of mitochondria. Here, we found that CML cells are vulnerable to DHODH inhibition mediated by Meds433, a new and potent DHODH inhibitor recently developed by our group. Meds433 significantly activates the apoptotic pathway and leads to the reduction of amino acids and induction of huge metabolic stress in CML CD34+ cells. Altogether, our study shows that DHODH inhibition is a promising approach for targeting CML stem/progenitor cells and may help more patients discontinue the therapy.

## Introduction

CML is a hematopoietic stem cell disorder emanating from a reciprocal translocation between ABL1 on chromosome 9 and BCR on chromosome 22. The encoded protein through constant tyrosine kinase activity triggers downstream signaling pathways and governs the expansion of leukemic cells [1]. The introduction of different generations of tyrosine kinase inhibitors (TKIs) has changed treatment outcomes and obtaining an operational cure is possible. However, not all patients fully benefit from TKI therapy, and TKI resistance and relapse after therapy discontinuation have been recorded. An alternative approach that can target CML clones irrespective of their state might help to increase the number of patients who can halt the therapy and have a better treatment outcome [2–7].

Progression and drug resistance of leukemic cells require rewiring in metabolic state and energy production. This adaption is managed by mitochondria as the pivotal metabolic organelle and as the main hub for ATP production, apoptosis, and synthesis of building blocks such as amino acids and nucleotides [8, 9]. Pyrimidine supply in resting and differentiated cells primarily relies on the salvage pyrimidine pathway that is energetically affordable. This level of nucleotide production for fast proliferating leukemic cells is insufficient and they have to fulfil their needs via the de novo pathway.

Dihydroorotate dehydrogenase (DHODH) is a vital enzyme in the de novo pyrimidine biosynthesis pathway and it is located in the inner mitochondria membrane. DHODH catalyzes the fourth step of the pyrimidine biosynthesis pathway by oxidation of dihydroorotate to orotate [10]. Several DHODH inhibitors such as Brequinar (BQ) have been employed to target this druggable enzyme in solid tumors but conducted clinical trials demonstrated little benefit for patients [10]. However, in 2016 Sykes et al. proved that BQ was able to unlock differentiation block in acute myeloid leukemia (AML) and managed to reduce tumor burden in vivo [11]. In contrast with AML, differentiation therapy in CML is not the main goal of treatment, and developing a more potent DHODH inhibitor that can eliminate leukemic cells while preserving its selectivity is of paramount importance.

Previously, a new and potent DHODH inhibitor called Meds433 was developed by our group [12–14]. In the present study, we shed light on the role of DHODH inhibition in CML stem/progenitor cells and how it can be a promising approach in the treatment of CML.

## Results

### DHODH inhibition induces apoptosis in CML

**Fig. 1:**
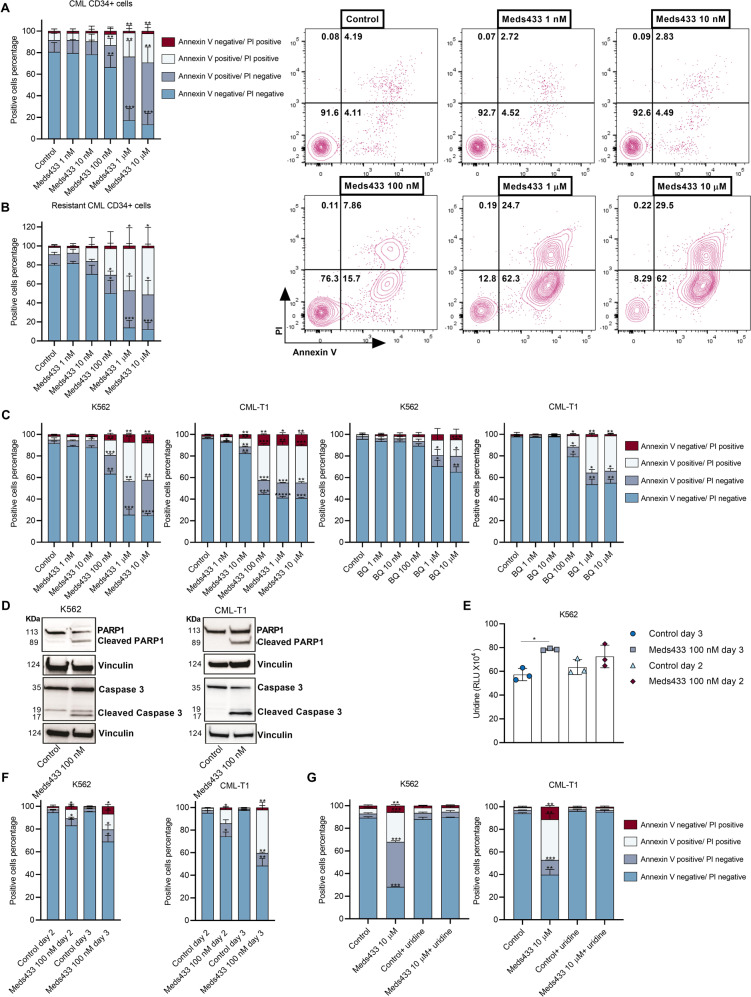
DHODH inhibition induces apoptosis in CML. **A** represents the positive cell percentage of Annexin V/PI subsets. The results were obtained in newly diagnosed CML CD34+ (n:6) patients’ cells after treatment with various concentrations of Meds433 for 3 days. Also, flow cytometry graphs of one treated CML CD34+ cells are shown in the **A** right panel. **B** represents the effect of Meds433 on resistant CML CD34+ cells (n:4) which is shown by the positive cell percentage of Annexin V/PI subsets. **C** demonstrates apoptosis induction that is shown by positive cells’ percentage of Annexin V/PI subsets after treatment of K562 and CML-T1 with different concentrations of Meds433 and BQ for 3 days (n:3). **D** shows Western blot analysis of PARP1 and Caspase 3 after treatment of K562 and CML-T1 with 100 nM Meds433 for 3 days (n:3). **E** shows uridine level based on RLU in the cell lysate of K562 cells after treatment with 100 nM Meds433 for 2 and 3 days (n:3). RLU inversely correlates with the uridine level in cells. **F** displays the kinetic of apoptosis after treatment of K562 and CML-T1 with 100 nM Meds433 (n:3). **G** represents uridine rescue that was measured using Annexin V/PI in K562 and CML-T1 after the addition of exogenous uridine at the concentration of 100 µM (n:3). The addition of uridine activates the salvage pyrimidine pathway and hampers activation of the de novo pyrimidine biosynthesis pathway. *p < 0.05, **p < 0.01, ***p < 0.001, ****p < 0.0001, *****p < 0.00001.

Previously it has been shown that pyrimidine starvation activates apoptotic pathways in different hematological malignancies, but its impact on CML cells was unknown. To study this, various concentrations (1 nM to 10 µM) of Meds433 for treatment of newly diagnosed (Fig. 1A) and resistant CML CD34+ cells (Fig. 1B) were used for 3 days (Flow cytometry graphs of one newly diagnosed patient sample treated with Meds433 are displayed in Fig. 1A right panel). The starting concentration that remarkably reduced cell viability in newly diagnosed and resistant CML stem/progenitor cells was 100 nM. Similarly, treatment of K562 and CML-T1, two different CML cell lines, with Meds433 followed the same pattern as CML primary cells in the induction of apoptosis (Fig. 1C). To compare the potency of Meds433 with BQ in the induction of apoptosis we measured the apoptotic effect of BQ on K562 and CML-T1 cells after 3 days of treatment (Fig. 1C). Based on the obtained results, BQ did not show any apoptotic effect at low concentrations and its activity was observed mainly at high concentrations from 1 to 10 µM. The half-maximal effective concentration (EC50) that induces apoptosis by Meds433 and BQ in CML cells is shown in Fig. 1SA. These results are in line with previous findings demonstrating that the half-maximal inhibitory concentration± standard error (IC50 ± SE) value of Meds433 and BQ in targeting pure DHODH enzyme activity is 1.2 ± 0.2 and 1.8 ± 0.8 nM, respectively [12]. These results prove the potency of our agent as a new generation of DHODH inhibitors. Also, the apoptotic effect of Meds433 on KU-812, JURL-MK1, and AR230-R (resistant to imatinib) cell lines is shown in Fig. 1SB.

To confirm the apoptosis induction, Caspase 3 and PARP1 were measured after the treatment of K562 and CML-T1 with 100 nM Meds433 for 3 days. The presence of cleaved PARP1 and cleaved Caspase 3 demonstrated the activation of the apoptotic pathways in CML cells treated with the DHODH inhibitor (Fig. 1D). (Uncropped images are available in the Supplemental File).

As targeting DHODH interferes with the pyrimidine biosynthesis pathway and to see if it affects the end product, uridine level in cell lysate was measured after 2 and 3 days of K562 treatment with 100 nM Meds433. As is shown in Fig. 1E, while the uridine level did not change after 2 days, it significantly decreased after 3 days of treatment (RLU inversely correlates with the uridine level in cells). To study if the change in the uridine level is the key element in the induction of apoptosis, a kinetic assay was performed in K562 and CML-T1. As is shown in Fig. 1F, apoptosis started after 2 days of Meds433 treatment and it significantly increased after 3 days.

To prove the selectivity of our agent in targeting DHODH, exogenous uridine was employed. As is shown in Fig. 1G, the addition of uridine to the culture media abolished the apoptotic effect of Meds433 at a high concentration of 10 µM after 3 days of treatment in K562 and CML-T1. The uridine rescue in two other CML cell lines is shown in Fig. 1SC.

### DHODH inhibition suppresses CML cell growth and induces cell cycle arrest

**Fig. 2:**
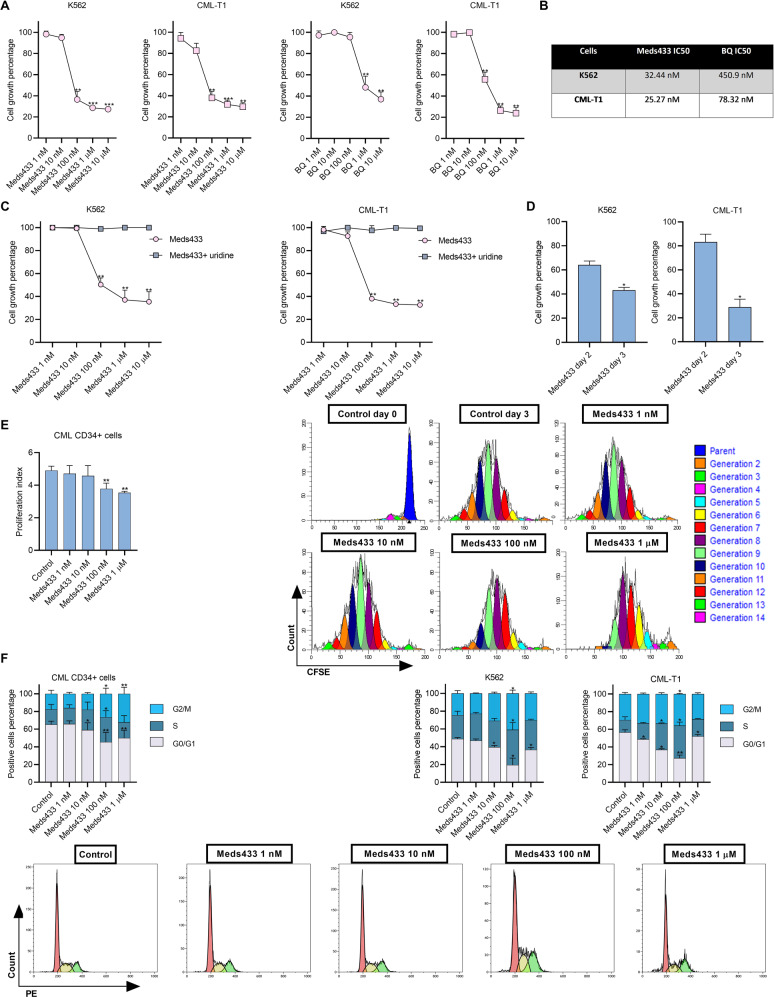
Effect of DHODH inhibition on cell cycle and cell growth rate. **A** displays cell growth rate by CCK8 assay in K562 and CML-T1 treated with Meds433 and BQ for 3 days (n:3). To calculate the cell growth rate, OD values were subtracted from the blank. Then, the OD values of treated cells were normalized to the OD values of the control group. **B** demonstrates IC50 of Meds433 and BQ in K562- and CML-T1-treated cells using cell growth data. **C** shows rescue of cell growth inhibition in CML cell lines in the presence of exogenous uridine at the concentration of 100 µM after 3 days of treatment (n:3). **D** displays kinetic of cell growth rate after the treatment of K562 and CML-T1 with 100 nM Meds433 obtained by CCK8 assay (n:3). **E** shows proliferation index in CML CD34+ cells (n:3) treated with different concentrations of Meds433 for 3 days and flow cytometry graphs of one treated patient (right panel). The proliferation index is considered as the total number of cell divisions divided by the number of cells that underwent division. **F** demonstrates cell cycle analysis in CML CD34+ cells (n:6), two different CML cell lines (n:3), and flow cytometry graphs for one patient CD34+ cells treated with Meds433 for 3 days. At a high concentration of 1 µM, the number of live cells was less than at the lower concentrations. This might cause a dissimilarity between the percentage of G1/S/G2M at the concentration of 1 µM in comparison to 100 nM Meds433. *p < 0.05, **p < 0.01. ***p < 0.001.

To see if pyrimidine starvation interferes with the cell growth of CML cells, the cell growth rate was measured in CML cell lines after 3 days of treatment with various concentrations of Meds433 and BQ. As is shown in Fig. 2A, treatment of K562 and CML-T1 with Meds433 and BQ hampered the cell growth rate. The starting concentration of Meds433 that reduced cell growth rate was 100 nM. As is shown in Fig. 2A, Meds433 displayed a higher potency compared to BQ and in K562, in fact, it reduced cell growth at one log lower concentration. Cell growth inhibition of KU-812 and JURL-MK1 by Meds433 is shown in Fig. 2SA. Also, IC50 of Meds433 and BQ that inhibits cell growth is shown in Fig. 2B.

To confirm if pyrimidine deprivation is the lead cause of cell growth inhibition, exogenous uridine was added to the culture media. As is shown in Fig. 2C, the addition of uridine to the culture media at the concentration of 100 µM fully rescued the inhibitory effect of Meds433 in K562 and CML-T1 treated cells. Meanwhile, the kinetics of cell growth in K562 and CML-T1 using 100 nM Meds433 followed the same pattern as apoptosis and a higher inhibitory effect was seen after 3 days of treatment (Fig. 2D).

To confirm these results in CML stem/progenitor cells, CML CD34+ were labeled with carboxyfluorescein succinimidyl ester (CFSE), and the proliferation index was measured by flow cytometry after 3 days of treatment with 100 nM Meds433. As is shown in Fig. 2E, Meds433 treatment could suppress the proliferation of CML CD34+ cells at 100 nM and 1 µM concentrations. Generated proliferation patterns of one newly diagnosed patient sample treated with Meds433 are shown in Fig. 2E.

This cell growth inhibition implied that we might have a cell cycle arrest in CML cells following DHODH inhibition. After the treatment of CML cells for 3 days with different concentrations of Meds433, the cell cycle was analyzed on the live cells population. As is displayed in Fig. 2F, treatment of CML CD34+ cells with different concentrations of Meds433 induced cell cycle arrest in the G2/M phase. Also, Meds433 induced G2/M arrest in K562, CML-T1 (Fig. 2F right panel), KU-812, and JURL-MK1 (Fig. 2SB) but the concentrations that caused this arrest were different. Many variables may lead to the subtle difference between Meds433 at a concentration of 100 nM and at a concentration of 1 µM in inducing cell cycle arrest, such as the timing of the experiment (72 h), cell viability, and the baseline rate of division of each cell type. Overall, Meds443 at 100 nM might be a relevant concentration for inducing a striking biological effect after 3 days of treatment.

Also, cell cycle patterns of one newly diagnosed patient sample treated with Meds433 are shown in Fig. 2F.

### Meds433 suppresses tumor growth in a CML xenograft model

**Fig. 3:**
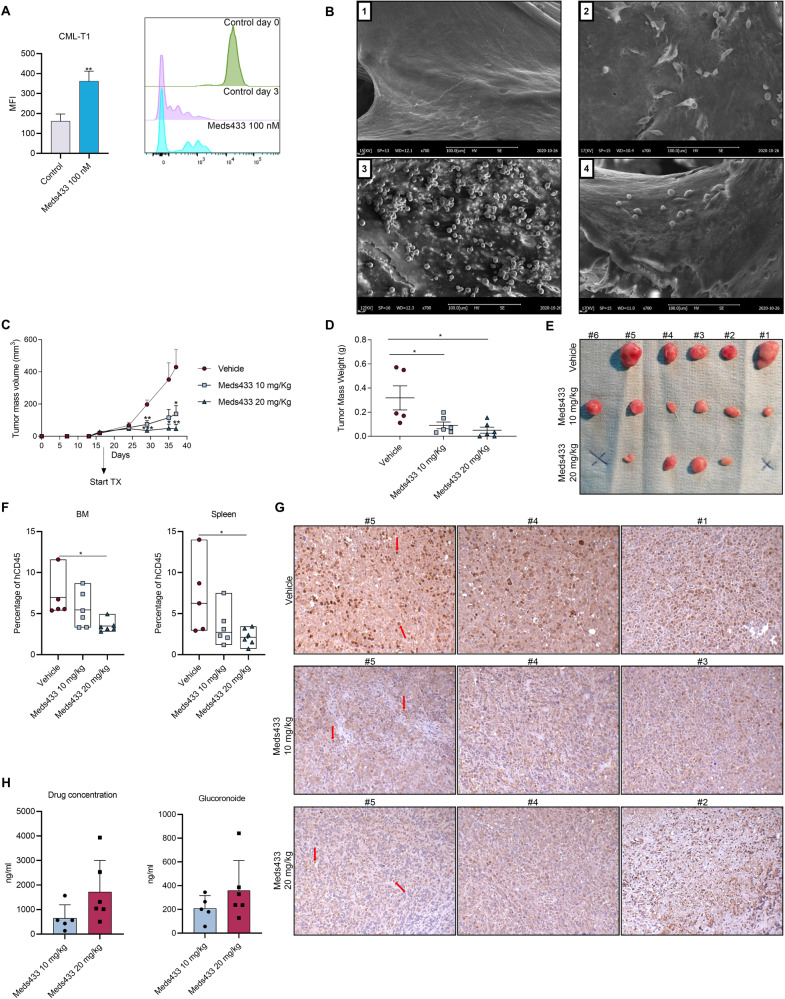
Meds433 impedes tumor growth in 3D co-culture and xenograft mice. **A** shows mean fluorescent intensity (MFI) of CFSE-labeled CML-T1 treated with 100 nM Meds433 for 3 days in a 3D co-culture platform (n:3) (a higher, mean fluorescent intensity, MFI, means that cells are non-proliferative while a lower MFI means that cells proliferate more and CFSE dye is divided between daughter cells). (B) demonstrates electron microscopy images of the 3D co-culture platform. **B1** shows demineralized bone matrix (DBM) treated with collagen type 1. **B2** shows mesenchymal stromal cells (MSCs) in the DBM scaffold. **B3** shows the addition of labeled CML-T1 with CFSE to the MSCs in the scaffold. **B4** displays a 3D co-culture condition treated with 100 nM Meds433. **C**, **D** represent KU-812-derived tumor mass volume and tumor weight after the treatment with 10 and 20 mg/kg Meds433, respectively. **E** represents the morphology of tumors treated with Meds433. Noteworthy, the administration of Meds433 20 mg/kg eliminated tumors in two mice. **F** shows tumor burden after the treatment with 10 and 20 mg/kg Meds433 in BM and spleen. **G** displays an immunohistochemical analysis of Ki67 expression in the vehicle, 10 mg/kg, and 20 mg/kg Meds433 treated mice (×20 magnification). Arrows indicate Ki67-positive cells and the number is related to tumors of each mouse displayed in **E**. **H** represents drug concentration and Glucoronoide as the drug metabolite in the plasma of treated mice. *p < 0.05, **p < 0.01, ***p < 0.001.

Before confirming the inhibitory effect of pyrimidine starvation on cell growth of CML cells in vivo, we designed a 3D co-culture platform to study the efficacy of our agent in targeting leukemic cells in a 3D complex microenvironment. Our results proved that treatment of CML-T1 co-cultured with stromal cells on a demineralized bone matrix (cancellous bone) scaffold with 100 nM Meds433 for 3 days reduced the proliferation of leukemic cells and impeded their expansion (mean fluorescent intensity, MFI, inversely correlates with the proliferation rate) (Fig. 3A). The surface electron microscopy (SEM) images of this 3D co-culture experiment are shown in Fig. 3B. SEM data revealed that CML-T1 colonies were eliminated after DHODH inhibition.

To demonstrate the efficacy of Meds433 in reducing cell growth of leukemic cells in vivo we used a xenograft mouse model of a human cell line (KU-812). Two doses of 10 and 20 mg/kg Meds433 were selected based on the maximum tolerated dose profile. Our results showed that administration of 10 mg/kg and 20 mg/kg Meds433 significantly reduced tumor mass volume compared to the vehicle group (Fig. 3C). Also, a reduction of the tumor weight was recorded in treated groups and confirmed the potency of Meds433 in controlling tumor growth (Fig. 3D). Tumor morphology is shown in Fig. 3E and demonstrates the reduction of tumor size in treated mice compared to the vehicle (H&E staining of tumors is displayed in Supplemental Fig. 3SA). One mouse in the vehicle group had to be sacrificed due to high tumor volume. Also, in two mice treated with 20 mg/kg of DHODH inhibitor, tumors were not detectable. To analyze tumor burden in mice, human CD45 was measured in the peripheral blood (PB), spleen, and bone marrow (BM). Based on our results we could not detect any tumor burden in PB (data not shown), while a small population of leukemic cells was detectable in the BM and spleen. Tumor burden was reduced significantly in mice treated with 20 mg/kg Meds433 (Fig. 3F). To confirm the inhibitory effect of Meds433 on tumor cell growth, immunohistochemical analysis for Ki67 expression, a nuclear marker linked to cell cycle and proliferation, was performed on tumors of three mice per group. As is shown in Fig. 3G, there was a significant reduction of Ki67 in 10 and 20 mg/kg Meds433-treated mice compared to the vehicle group. Also, at the end of the experiments, drug concentration and drug metabolite were measured in plasma of 10 and 20 mg/kg Meds433-treated mice (Fig. 3H). These results show that the amount of Meds433 in plasma grows up in a dose-dependent manner like its major metabolite, glucuronidated derivative, which represents 10–15% of total Med433 at the end of the experiment.

### Meds433 significantly changes transcriptome in CML CD34+ cells

**Fig. 4:**
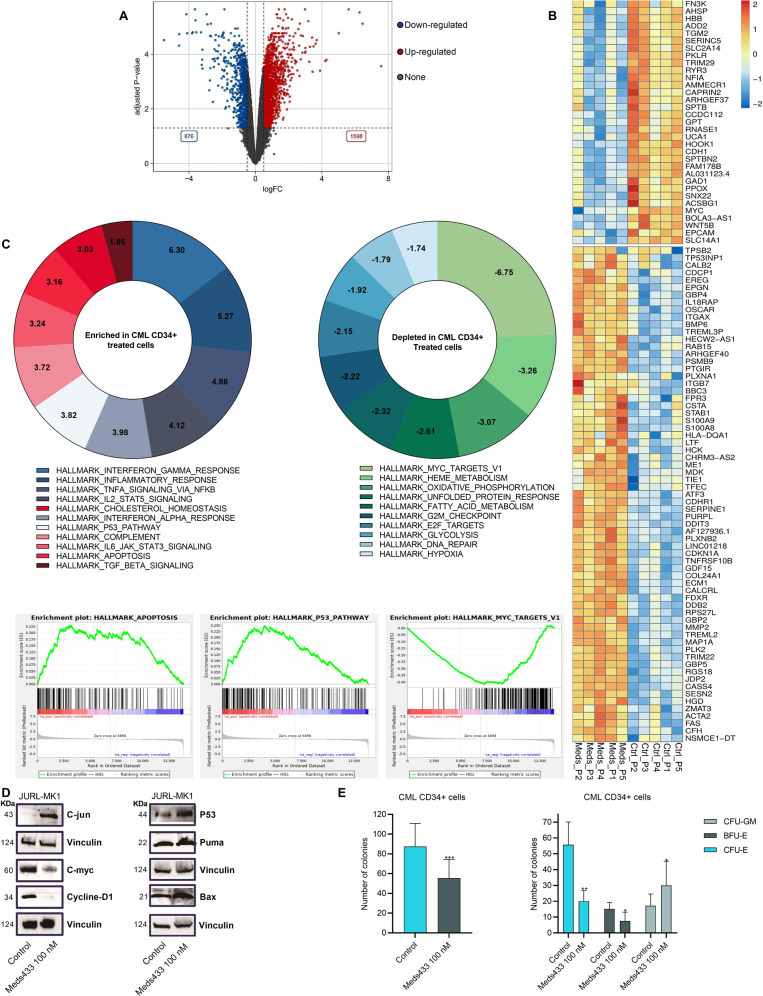
RNA-seq analysis following DHODH inhibition in CML. **A** The volcano plot demonstrates the number of up- and down-regulated genes in CML CD34+ (n:5) patients’ cells after treatment with 100 nM Meds433 for 3 days. **B** The top 100 differentially expressed genes based on adjusted *p* value are depicted in the heatmap, and **C** shows enriched and depleted hallmark gene sets and three enrichment plots related to apoptosis, P53 pathway, and MYC targets. The number in the plots demonstrates a normalized enrichment score (NES). **D** demonstrates Western blot analysis of JURL-MK1 cell line treated with 100 nM Meds433 for 3 days (n:3). **E** shows CFU assay after the treatment of CML CD34+ (n:5) patients’ cells with Meds433. For this figure, the total number of colonies (CFU-GM + CFU-E + BFU-E) and the number of each subset are displayed. *p < 0.05, **p < 0.01, ***p < 0.001.

To study the effect of DHODH inhibition on transcriptome profiling of CML cells, RNA-seq was performed after treatment of CML CD34+ cells with 100 nM Meds433 for 3 days. By considering 0.5-log-fold expression level and FDR < 0.05 as the cutoff for both up and down-regulated genes, we observed that the number of upregulated genes (1598 genes) was higher compared to the down-regulated ones (876 genes) (Fig. 4A). Considering the top 100 differentially expressed genes (in terms of *p* value), upregulated genes were mostly related to apoptosis, movement, and differentiation toward myeloid lineage. Genes related to erythropoiesis, proliferation, and metabolism were downregulated after treatment (Fig. 4B). Then gene set enrichment analysis (GSEA) was performed to reveal gene sets that were enriched or depleted after treatment with Meds433 (Fig. 4C). The significantly enriched hallmark gene sets were mainly related to inflammation (such as interferon-γ, interferon-α, TNF-α, TGF-β), survival pathways (such as apoptosis, P53 pathway), and immune response (such as IL2-STAT5, complement). On the other hand, gene sets germane to cell cycle and proliferation (such as MYC targets, E2F targets, G2/M checkpoints), metabolism (such as heme metabolism, oxidative phosphorylation, fatty acid metabolism, glycolysis) were depleted. The validation by qRT-PCR confirmed some differentially expressed genes (Fig. 4SA).

Western blot analysis on JURL-MK1 confirmed alterations in known genes previously reported to be regulated by other DHODH inhibitors (Fig. 4D). An increase in C-jun, P53, Puma, and Bax and a decrease in C-myc and Cyclin-D1 were recorded after Meds433 treatment. The obtained results are in line with RNA-seq data and previous findings. (Uncropped images are available in the Supplemental File).

Previous studies showed that AML cells following pyrimidine deprivation exit the cell cycle and differentiate toward granulocyte/monocyte lineage. To confirm the alteration of lineage-specific genes after targeting DHODH in CML CD34+ cells, a colony-forming unit (CFU) assay was performed. This experiment reflects the potential of individual cells in proliferating and differentiating into different lineages. Our results showed that the total number of colonies (colony-forming unit—granulocyte/macrophage (CFU-GM) + colony-forming unit—erythroid (CFU-E) + burst forming unit—erythroid (BFU-E)) significantly decreased in CML CD34+-treated cells with 100 nM Meds433 (Fig. 4E, left panel). However, we found that this decrease was related to CFU-E and BFU-E potential (Fig. 4E, right panel). This reduction is related to the negative effect of DHODH inhibition on genes involved in erythropoiesis. Also, we had an increase in CFU-GM (Fig. 4E, right panel), which proves the tendency of a cell with stem cell potential to differentiate toward myelomonocytic lineage under stress conditions that in our case were caused by pyrimidine starvation.

### DHODH inhibition increases maturation markers and disrupts the normal function of mitochondria

**Fig. 5:**
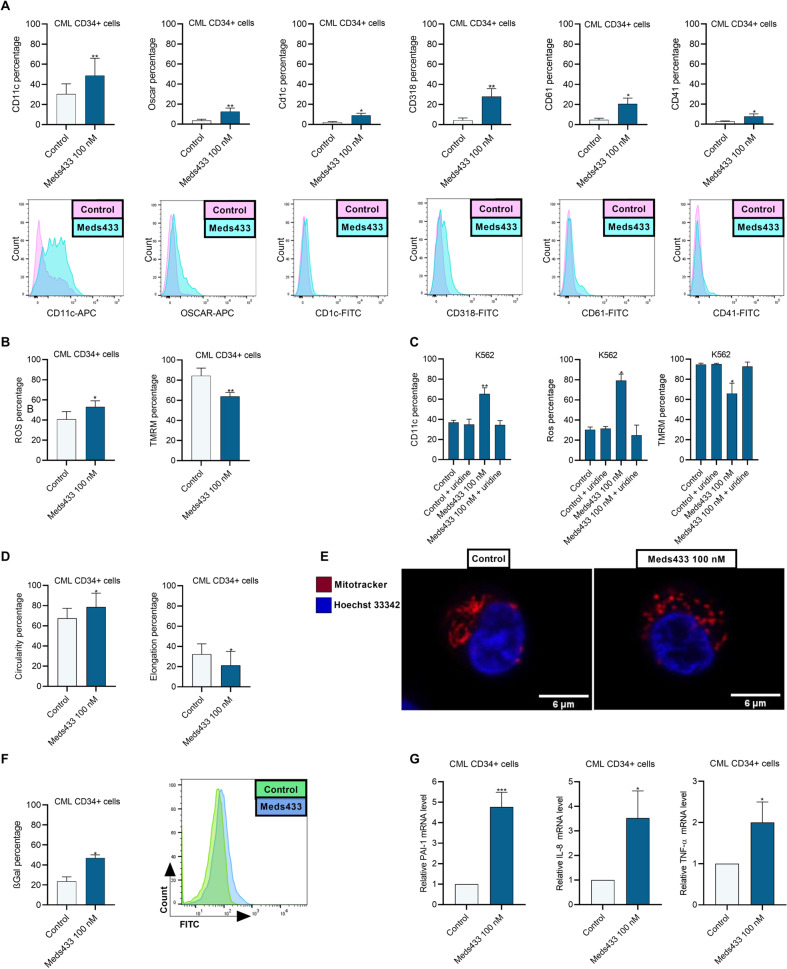
DHODH inhibition induces differentiation and disrupts mitochondria function in CML. **A** shows the percentage of lineage-specific CD markers on gated live cells after the treatment of CML CD34+ (n:21) patients’ cells with Meds433 for 3 days. Also, flow cytometry graphs of each CD marker are shown below each bar chart. **B** demonstrates measurement of ROS and mitochondria cell membrane potential using flow cytometry analysis in CML CD34+ (n:5) patients’ cells treated with Meds433 for 3 days. **C** represents CD11c percentage, ROS production, and mitochondria membrane potential after the addition of exogenous uridine in K562 treated cells after 3 days of treatment (n:3). **D** shows percentage of mitochondria with circularity>0.8 and elongation>1.25 in CML CD34+ (n:4) patients’ cells after 3 days of treatment with 100 nM Meds433. Inverse circularity was used as a measure of mitochondrial elongation. A value of circularity = 0.8 was chosen as the threshold for circular mitochondria and elongation = 1.25 was chosen as the threshold for elongated mitochondria. The graph shows the percentages of circular mitochondria (with circularity >0.8) and elongated mitochondria (elongation > 1.25) induced by our treatment. **E** displays confocal images of one CML CD34+ sample treated with 100 nM Meds433 for 3 days, where the nucleus was labeled with Hoechst dye (blue) and mitochondria were labeled with Mitotracker (red). **F** shows the β-Gal percentage in CML CD34+ (n:3) patients’ cells measured by flow cytometry after DHODH inhibition for 3 days. For β-Gal analysis, live cells were gated. Also, a flow cytometry graph of one treated patient sample is shown. **G** demonstrates relative mRNA levels of PAI-1, IL-8, and TNF-α in treated CML CD34+ (n:3) with 100 nM Meds433 for 3 days. *p < 0.05, **p < 0.01, ***p < 0.001.

To validate the result of RNA-seq data and CFU assay in inducing differentiation following DHODH targeting, lineage-specific cell surface markers were measured by flow cytometry analysis. After the treatment of CML CD34+ cells with 100 nM Meds433 for 3 days, we found a significant increase in CD11c, OSCAR, CD1c (markers of dendritic cell and monocyte lineage), CD318 (a marker of cell migration), and CD61, CD41 (megakaryocytic markers) (Fig. 5A). Also, flow cytometry graphs of each CD marker are shown below each bar chart.

As DHODH is placed in the inner membrane of mitochondria, targeting this enzyme might affect mitochondria function. To confirm this, we showed that DHODH inhibition in CML CD34+ cells increased ROS production and reduced mitochondria cell membrane potential after 3 days of Meds433 treatment (Fig. 5B). This phenomenon is known to eventually result in mitochondria damage and subsequent apoptosis. As is shown in Fig. 5C, exogenous uridine rescued the effect of DHODH inhibition in the enhancement of CD11c, ROS production, and reduction of mitochondria membrane potential in K562 cells.

To demonstrate the role of DHODH inhibition on mitochondria morphology we analyzed the structural features of mitochondria by confocal microscopy. Our results showed that DHODH inhibition in CML CD34+ cells for 3 days is concomitant with the reduction of elongation and enhancement of circularity in mitochondria (Fig. 5D). Representative confocal images of one CML CD34+ sample treated with 100 nM Meds433 are displayed in Fig. 5E.

Inflammation profile, cell cycle arrest, cell growth arrest, and ROS production implied that treated CML CD34+ cells might undergo senescence and lose their self-renewal capability. To prove this, we measured β-galactosidase (β-Gal) in CML CD34+-treated with 100 nM Meds433 for 3 days, and a significant increase in β-Gal percentage was recorded (Fig. 5F). To confirm the senescence phenotype in CML CD34+-treated cells, some genes belonging to the senescence-associated secretory phenotype (SASP) such as PAI-1, IL-8, and TNF-α were measured by qRT-PCR. As is shown in Fig. 5G, an increase in these genes confirmed the results of flow cytometry analysis in which Meds433 was able to induce senescence in CML CD34+ cells.

### Targeting DHODH alters metabolic profile in CML stem/progenitor cells

**Fig. 6:**
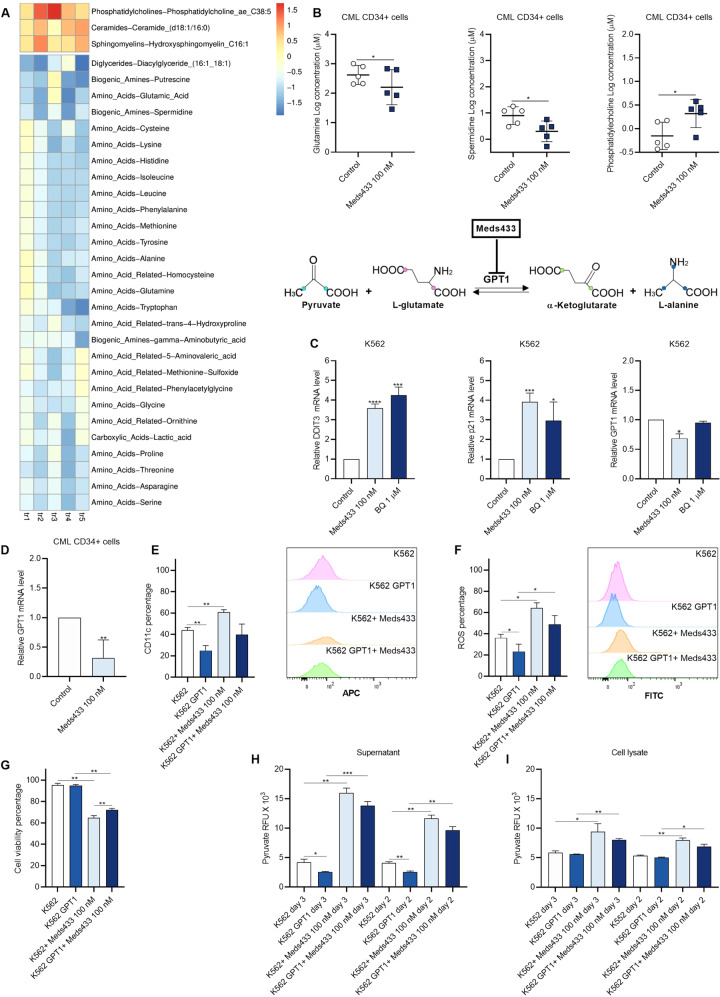
DHODH inhibition changes the metabolic profile in CML CD34+ cells. **A** shows altered metabolites following treatment of CML CD34+ (n:5) with Meds433 for 3 days. **B** represents the micromolar concentration of some metabolites in treated and untreated CML CD34+ cells (these metabolites were selected based on their relevance to different biological effects caused by Meds433). **C** shows the relative mRNA level of DDIT3, p21, and GPT1 in K562 cells treated with 100 nM Meds433 and 1 µM BQ for 3 days (n:3). **D** shows the relative mRNA level of GPT1 in treated CML CD34+ (n:5) patients’ cells for 3 days. **E**–**G** show the percentage of CD11c (n:4), ROS (n:4) production, and cell viability (n:3) in K562 and GPT1 overexpressed K562 after 3 days, respectively. Also, corresponding flow cytometry graphs of CD11c and ROS are shown. **H**, **I** demonstrate Pyruvate levels based on RFU in the supernatant and cell lysate of K562 and GPT1 overexpressed K562 after 2 and 3 days of the treatment, respectively. In **E**, **F**, **H**, and **I**, K562- and GPT1-treated Meds433 were not compared due to different levels of untreated groups. *p < 0.05, **p < 0.01, ***p < 0.001.

To demonstrate if targeting DHODH activity could alter the cellular metabolism, 630 metabolites were quantified after treatment of CML CD34+ cells with 100 nM Meds433 for 3 days. Our results showed that amino acids decreased after treatment with Meds433 (Fig. 6A). Among these amino acids, glutamine is of high importance as it is a key component of the pyrimidine biosynthesis pathway. On the other hand, an increase in phosphatidylcholines, ceramides, and sphingomyelins that have a role in apoptosis, differentiation, and senescence in treated cells is in line with previous findings (Fig. 6A). The concentrations of glutamine, spermidine, and phosphatidylcholine in treated and untreated CML CD34+ are shown in Fig. 6B. The concentration of some other metabolites after the treatment of CML CD34+ cells with Meds433 is shown in Fig. 5SA.

Moreover, we found that glutamic pyruvic transaminase (GPT1, GPT) is among the top downregulated genes (Fig. 4B) after the treatment of CML CD34+ cells with 100 nM Meds433. GPT1 is an enzyme that catalyzes the reversible transamination from alanine and α-ketoglutarate to produce pyruvate and glutamate, thus, it plays a major role in amino acids and glucose metabolism. First, we compared the effect of BQ on some up and down-regulated genes to see if it can modulate the expression level of different genes in a similar way to Meds433. To do so, we treated K562 cells with 100 nM Meds433 and 1 µM BQ (the concentration in which BQ showed the biological effects in K562 cells) for 3 days and expression of GPT1 and two other known genes relevant to cell cycle and apoptosis such as p21 and DDIT3 was measured by qRT-PCR. Our results showed that while both Meds433 and BQ enhanced the expression of p21 and DDIT3, BQ did not impact GPT1 expression and the downregulation was specific for Meds433 treated K562 (Fig. 6C). To confirm the inhibitory effect of Meds433 on GPT1, the mRNA level of GPT1 in CML CD34+-treated cells was measured and a significant down-modulation was seen (Fig. 6D). To clarify the role of GPT1 in CML cells, we overexpressed GPT1 in K562 using lentiviral particles (data not shown) and we studied the relationship between this gene and some biological effects caused by Meds433. As is shown in Fig. 6E, F, treatment of K562 with Meds433 increased the expression of CD11c level and ROS production. However, CD11c expression and ROS level significantly decreased in GPT1 overexpressed K562 compared to K562 cells. In addition, the level of cell viability in GPT1 overexpressed K562 treated with Meds433 cells was significantly higher than treated with K562 cells (Fig. 6G). Meanwhile, to confirm the metabolic alteration after the change in GPT1 expression, pyruvate level in the supernatant and cell lysate of K562-treated and control cells based on the relative fluorescent unit (RFU) was measured. Our results showed that pyruvate significantly increased after Meds433 treatment in both supernatant (Fig. 6H) and cell lysate (Fig. 6I) of K562-treated cells. Interestingly, the level of pyruvate in the supernatant of GPT1 overexpressed K562 was lower than K562.

As is shown in Fig. 6 overexpression of GPT1 in K562 cells had opposite effects on CD11c, ROS, cell viability, and pyruvate level (supernatant) compared to Meds433. This might show that one of the possible mechanisms in which Meds433 is effective in CML is through the downregulation of GPT1.

## Discussion

About 40–50% of the newly diagnosed CML patients are eligible to stop TKIs, though half of them will relapse within 6–12 months from the therapy discontinuation. This finding confirms that cells that have the biological potential to cause leukemia are surviving and resisted TKI therapy. Therefore, targeting biological differences between normal and leukemic cells other than BCR-ABL1 might boost the number of patients attaining TFR [15–17]. One of the differences between normal and leukemic cells is the metabolic reprogramming of leukemic cells that provides enough nucleotides and amino acids for their progression [18]. In this study Meds433, as a potent DHODH inhibitor, was used to demonstrate the effect of pyrimidine deprivation in targeting CML stem/progenitor cells.

Previously, it has been reported that inhibition of DHODH in different hematological malignancies induces apoptosis and differentiation of the targeted cells [19–24]. The predominance of each mechanism might be context-dependent or it might be related to the potency of a DHODH inhibitor. Our results demonstrated that blocking the pyrimidine biosynthesis pathway in CML CD34+ cells of newly diagnosed and resistant patients induces apoptosis. Our data suggest a direct relation between nucleotide deprivation and activation of apoptosis in CML. This also proves the activity of the de novo pyrimidine biosynthesis in CML cells and their high demands for nucleotides. It has been shown that pyrimidine deprivation results in upregulation and activation of wild-type P53 [25, 26]. Our data showed that DHODH inhibition significantly suppressed CML cell growth in vitro and in vivo and induced G2/M arrest. Based on the RNA seq data, this effect might be mediated through the reduction of a proto-oncogene such as MYC and upregulation of tumor suppressors such as P53 and DDIT3. Previous findings claimed that upregulation of P53 and downregulation of MYC, suppress cell growth, and induce cell cycle arrest [27]. Apart from the role in apoptosis and proliferation, MYC also regulates cell metabolism through direct upregulation of DHODH and an increase in glutamine transporter [28–30]. Glutamine is necessary for the *de novo* pyrimidine biosynthesis pathway and our study showed that DHODH inhibition led to the reduction of most amino acids including glutamine. This depletion might occur directly through the detrimental effect of DHODH inhibition on amino acid transporters, amino acid synthesis pathways, or indirectly via reduction of MYC. It looks like targeting DHODH activity in CML destroys all the building blocks for leukemic progression and induces significant metabolic stress in cells. This metabolic stress results from amino acid deprivation simultaneously activating P53 and causing cell cycle arrest [10, 31, 32]. Furthermore, amino acids such as glutamate, glycine, and cysteine are necessary for the synthesis of glutathione and the maintenance of redox balance [33]. The disruption in redox balance increases ROS production and decreases mitochondria membrane potential, as we saw in treated CML CD34+ cells [34]. Also, reduction of erythroid lineage after treatment with Meds433 might suggest the role of DHODH in erythropoiesis. It has been reported that nucleotide production by glutamine has an essential role in erythroid lineage commitment, and a disruption in this pathway shifts the hematopoietic stem cells toward myelomonocytic lineage [35]. The reduction of erythroid genes and erythroid colonies after the treatment with Meds433 suggests the essential role of nucleotides in cell fate decisions. Moreover, the inflammatory profile caused by DHODH inhibition has a negative impact on the erythroid commitment [36] and promotes myelomonocytic differentiation.

Our data showed that while both Meds433 and BQ target DHODH enzyme, not all up and downregulated genes are similarly affected. In our study, we found that Meds433 down-modulated expression of GPT1 in CML CD34+ cells and K562 cells. Previous studies confirmed the role of this enzyme in various cancers and one study reported that targeting GPT1 could reduce the growth of hepatocellular carcinoma [37–39]. Our overexpression data showed that Meds433 might act also through this peculiar metabolic pathway, as opposed to other DHODH inhibitors

On balance, our study shows that the *de novo* pyrimidine biosynthesis pathway is active in CML cells which makes them vulnerable to its inhibition. Meds433 as a potent DHODH inhibitor depletes nucleotides production that leads to amino acid deprivation, induction of metabolic stress, and activation of apoptotic pathways in leukemic cells.

## Materials and methods

### CML sample collection

For this study, 60 BM and PB samples of newly diagnosed and 4 TKI-resistant CML patients were collected as part of the Italian national study GIMEMA CML1415, approved by the Ethical Committee of the Coordinating Centers on 11 May 2016. Informed consent was obtained from all patients.

### Cell culture

For ex vivo study, mononuclear cells (MNCs) were isolated from both BM and PB using Ficoll-Hypaque (Sigma Aldrich, Milan, Italy). Then, CML CD34+ cells were positively isolated by immunomagnetic separation (MACS, Miltenyi Biotec, Italy) and cultured in serum-free stemMACS media (Miltenyi Biotec, Italy) with recombinant human Flt3L (100 ng/mL), SCF (100 ng/mL), IL3 (20 ng/mL), and TPO (20 ng/mL). All of the ex vivo experiments were performed on CML CD34+ population as CML stem/progenitor cells.

The BCR-ABL-positive cell lines K562 (ATCC), KU-812 (ATCC), JURL-MK1 (DSMZ), and CML-T1 (DSMZ) were maintained in RPMI 1640 (Gibco, Thermo Fisher Scientific) supplemented with 10% heat-inactivated fetal bovine serum (FBS, Gibco, Thermo Fisher Scientific). The imatinib-resistant AR230R (ATCC) cell line was cultured in the presence of 1 μM imatinib. The human mesenchymal stromal cells HS-5 (ATCC) were cultured in DMEM (Microtech, Naples, Italy) supplemented with 10% FBS. Media were supplemented with 1% penicillin/streptomycin (Gibco, Thermo Fisher Scientific). Cell lines were maintained in culture for no longer than 5–6 weeks and were routinely tested for mycoplasma contamination.

For treatment, CML CD34+ and CML cell lines were seeded 10,000 cells (50,000 cell/ml) in 96-well plate and incubated at 37 °C with Meds433 or BQ, or DMSO, as the vehicle, for 48 or 72 h at the indicated concentrations.

### Annexin V/PI staining

After treatment of CML CD34+ cells with Meds433 from 1 nM to 10 µM and CML cell lines with Meds433 and BQ from 1 nM to 10 µM, the percentages of viable cells were determined by flow cytometry analyses using Annexin V-FITC Kit (Miltenyi Biotec, Italy), according to the manufacturer’s instructions. This kit allows us to detect the percentage of viable, apoptotic, and necrotic cells. After the PI addition, samples were acquired on FACSVerse and analyzed by Kaluza software version 2.1 (Beckman Coulter Fullerton, CA). To study the selectivity of Meds433, exogenous uridine 100 µM was added to the culture media of four CML cell lines treated with 10 µM Meds433 and Annexin V/PI was assessed.

### 3D co-culture

Demineralized bone matrix (DBM, Orthosis Block Geistlich Biomaterial Italia) was coated with collagen type I (100 µg/mL) overnight at 4 °C. The coated scaffolds were washed with PBS and centrifuged at 900 g for 5 min to unblock the surface pores. in all, 1 × 10^5^ HS-5 cells were seeded and cultured in DMEM 10% FBS for 24 h. After a wash with PBS, 1 × 10^5^ CML-T1 cells labeled CFSE were added to the scaffold in IMDM with 10% FBS. After 24 h, the scaffolds were washed with PBS to remove unattached cells. Then the scaffolds were treated with 100 nM Meds433 for 3 days and the mean fluorescent intensity of CML-T1-labeled CFSE and morphology by the surface electron microscopy was measured.

### CML xenograft mouse model

KU-812 cells (1 × 10^7^ cells in PBS mixed 50:50 with Matrigel™ (BD, Bioscience) were implanted subcutaneously into the left flank of 8-week-old NOD/SCID/γ chain−/− (NSG) immunocompromised male mice. Mice were treated with vehicle or Meds433 without randomization when the volume of masses reached approximately 0.2 cm^3^ and was palpable. Mice were treated intraperitoneal for 21 consecutive days with vehicle (n:6) (5% DMSO and 95% corn oil from Sigma Aldrich), 10 mg/kg Meds433 (n:6), and 20 mg/kg (n:6). The tumor volume was measured once a week with a caliper and calculated with *V* = 1/2 × (length × width^2^). Mice were treated following the European guidelines and with the approval of the Italian Ministry of Health (Authorization n. 42/2020-PR). Mice were euthanized when the dimension of tumors in the control group exceeded the size of 1.5 cm^3^. At the end of the experiment, PB, BM, and spleen were collected to evaluate tumor burden by flow cytometry, using hCD45+ (#62-9459-42) (Invitrogen, Life Technologies). Also, hematoxylin and eosin staining of the tumor xenografts was performed.

### RNA sequencing

Gene expression profiling was performed on CD34+ cells from five CML patients, untreated, and after 3 days of treatment with 100 nM Meds433, for a total of 10 matched samples. Total RNA was isolated using TRIzol reagent (Invitrogen), according to the manufacturer’s protocol. The quantity and quality of the starting RNA were checked by Qubit and Bioanalyzer (Agilent). Libraries were prepared using the TruSeq Stranded mRNA Sample Prep Kit (Illumina) following the manufacturer’s instructions. Libraries were sequenced on Illumina NextSeq 500 System (single-end 75 bp reads). Sequencing reads were aligned to the human reference genome (version GRCh38.p13) using STAR v2.7.7a0 [40] (with parameters –outFilterMismatchNmax 999 --outFilterMismatchNoverLmax 0.04) and providing a list of known splice sites extracted from GENCODE comprehensive annotation (version 32). Gene expression levels were quantified with featureCounts v1.6.3 [41] (options: -t exon -g gene_name) using GENCODE gene annotation (version 32 basic). Multi-mapped reads were excluded from quantification. Gene expression counts were next analyzed using the edgeR package [42]. Normalization factors were calculated using the trimmed-mean of *M*-values (TMM) method (implemented in the calcNormFactors function) and RPKM was obtained using normalized library sizes and gene lengths. After filtering lowly expressed genes (below 1 count per million mapped reads (CPM) in 6 or more samples), differential expression analysis was carried out by fitting a quasi-likelihood negative binomial generalized log-linear model using the patient’s identity as a blocking factor and performing QLF test comparing treated and untreated samples. Genes were considered as significantly differentially expressed (DEGs) when having |logFC| > 0.5 and FDR < 0.05.

Reads per kilobase of transcript per million mapped reads (RPKM) values were scaled as Z-scores across samples before computing distances.

Gene Set Enrichment Analysis was performed using GSEA (v 3.0) in pre-rank mode using logFC obtained from the edger model as ranking score. Gene sets in the Molecular Signature Database were considered (v7.0), excluding sets bigger than 2500 and smaller than 5 genes. RNA sequencing data have been deposited to a public database with accession number: GSE177485.

### CFU assay

Primary CML CD34+ cells were cultured for 3 days with 100 nM Meds433 or control and then 500 cells/dish were suspended in IMDM supplemented with 2% FBS. Cells were plated in methylcellulose-containing medium (StemCell Technologies) in 35-mm dishes based on the manufacturer’s instruction and colonies were scored and counted after 15 days.

### Mitochondria circularity and elongation

Following the CML CD34^+^ culture without or with 100 nM Meds433, the image acquisition was performed with a Leica TCS SP5 confocal system (Leica Microsystems) equipped with an HCX PL APO ×63/1.4 NA oil-immersion objective. Images were acquired on the three coordinates of the space (XYZ planes) with a resolution of 70 nm × 70 nm × 300 nm at 8–12 cells/condition. To quantify mitochondria circularity and elongation, an ImageJ (Rasband, W.S., U.S. National Institutes of Health, Bethesda, MA) macro was created. Briefly, images were pre-processed with background subtraction and Gaussian blur and segmented using intensity thresholding; binary images were post-processed to improve mitochondrial detection, finally, the circularity was measured:

circularity = 4pi(area/perimeter^2).

### Metabolic profile analysis

A targeted metabolic profile was measured by the MxP® Quant 500 kit (Biocrates Life Sciences AG, Innsbruck, Austria). This kit measures 630 metabolites from 26 biochemical classes. The metabolites were extracted from CML CD34+ cells treated with 100 nM Meds433 for 3 days. Briefly, a 96-well-based sample preparation device was used to quantitatively analyze the metabolite profile in the CML CD34+ samples. This device consists of inserts that have been impregnated with internal standards, and a predefined sample amount was added to the inserts. Next, a phenyl isothiocyanate (PITC) solution was added to derivatize some of the analytes (e.g., amino acids), and after derivatization was completed, the target analytes were extracted with an organic solvent, followed by a dilution step. The obtained extracts were then analyzed by FIA-MS/MS and LC-MS/MS methods using multiple reaction monitoring (MRM) to detect the analytes. Concentrations were calculated using appropriate mass spectrometry software (Sciex Analyst) and normalized as picomoles per million cells. Further data analysis has been performed using MetaboAnalyst (version 5.0). Out of 630 features, 435 have a constant or single value across all 10 analyzed samples and were removed, 54 were removed because under the level of detection in more than 60% of samples. Missing variables were replaced by LoDs (1/5 of the min positive value for each variable). Permutation test on orthogonal projections to latent structures discriminant analysis (OPLS-DA) shows that samples can be correctly classified as treated or controls according to metabolites concentrations (Q2 Pvalue = 0.025, R2Y Pvalue = 0.053 over 1000 permutations). Data were normalized using a Probabilistic Quotient Normalization using control patients as reference group [43], log10 transformed and Pareto scaled.

In the heatmap in Fig. 6A, the metabolites with false discovery rate (FDR) < 0.1 (paired *t* test) are reported, the represented values are log fold change concentrations between treated and relative control samples. Among many significantly increased phosphatidylcholines detected, only one is reported as representative of the entire metabolite class.

### Statistical analysis

All data are reported as the mean ± standard error of the mean (SD) with ≥3 experiments in primary cells and cell lines. Experiments (in vitro, ex vivo) involving cells treated with DMSO (controls) or different Meds433 concentrations are based on paired samples, i.e., the same sample was analyzed under different conditions; accordingly, the statistical analyses utilized were a paired *t* test. For in vivo experiments, an unpaired *t* test was applied. Statistical analyses were obtained using GraphPad Prism version 8 (GraphPad Software, San Diego, CA). All tests were considered significant at *p* < 0.05. For the determination of EC50 and IC50, a nonlinear regression model was applied. For most of the in vivo and in vitro experiments and data analysis, at least two different operators were involved.

## Supplementary information

Supplemental material

Uncropped WB

Checklist

## Data Availability

The data used to support the findings of this study are available from the corresponding author upon request.
