# Supplementary material for: Dihydroorotate dehydrogenase inhibition reveals metabolic vulnerability in chronic myeloid leukemia
Source: Cell Death Dis. 2022 Jun 30;13(6):576. doi: 10.1038/s41419-022-05028-9 (PMC9247109; doi:10.1038/s41419-022-05028-9)
Supplement: Supplementary file 1 — Supplemental material [file 41419_2022_5028_MOESM1_ESM.doc]

**Supplemental data**

**Material and Methods:**

**Reagents**

Meds433 and Brequinar (BQ) were synthesized as described in (1). Reagents were dissolved in DMSO and diluted in culture media before use. The final DMSO concentration did not exceed 0.1%. Imatinib, uridine, and 2′,7′-Dichlorofluorescin diacetate (DCFDA) were purchased from Merck (Milan, Italy). Cytokines were purchased from Miltenyi Biotec, Italy.

**Protein preparation and Immunoblotting**

Total protein extracts were obtained by lysing cells using a buffer containing 150 mM NaCl, 1 mM EDTA, 50 mM HEPES pH 7.5, 1% Triton X-100, 10% Glycerol (Sigma-Aldrich) supplemented with protease and phosphatase inhibitors cocktail. After the lysis, cells were centrifuged at 14000 rpm for 15 min. Protein concentration was evaluated using BCA Reagent (Sigma-Aldrich) concentrate assay. Western blotting (WB) was performed separating 40 µg of proteins by SDS polyacrylamide gel electrophoresis (PAGE) and electroblotting onto nitrocellulose membranes (BioRad). Membranes were blocked in 5% non-fat milk PBS-Tween 0.1% buffer for 1 h at 37 °C, then incubated with appropriate primary and secondary antibodies in PBS-Tween 0.1% buffer, respectively, overnight at 4 °C or for 1 h at room temperature and developed using Chemidoc Touch Imaging System (Bio Rad). The following primary antibodies were used: anti-C-myc (sc-40) anti-C-jun (sc-1694) from Santa Cruz, anti-Bax (#2772), anti-Puma (#4976), anti-Cyclin-D1 (#2978), anti-PARP1 (#9542), and anti-Caspase 3 (#9662) (Cell Signaling Technology), anti-P53 (AF1355) from R&D Systems. Normalization was performed on Vinculin or Tubulin loading control levels.

**Determination of Pyruvate and Uridine triphosphate**

Pyruvate and uridine triphosphate levels were determined after 48h and 72h of treatment with 100 nM Meds433. At each time point, K562 cells were harvested and the metabolites contents were measured in the culture supernatant and/or cell lysate. Pyruvate contents were measured by fluorometric assay kit (Merck. #332) in both supernatant and cell lysate according to the manufacturer's protocol. The fluorescence was detected using Promega Microplate Reader (Glomax) with a 530 nm excitation and a 585 nm emission filter. Data are presented as Relative Fluorescence Units (RFU). The uridine triphosphate in cell lysate was measured by a chemiluminescent assay kit (Abbexa, CliniSciences, Nanterre, France), according to the manufacturer's protocol. Data are presented as Relative Light Units (RLU) and are inversely correlated to the concentration of uridine triphosphate.

**Cell growth assay**

The cell growth rate in CML cell lines treated with Meds433 or BQ was measured with Cell Counting Kit-8 (CCK-8, Microtech, Naples, Italy) according to the manufacturer's instructions. Briefly, CML cell lines were cultured with Meds433 or BQ from 1 nM to 10 µM and after two or three days, 10 µL of CCK-8 solution was added into each well and incubated for 2 hours. Optical density values were recorded using a plate reader at 450 nm, using a Promega Microplate Reader (Glomax). The cell growth rate of K562 and CMLT-1 cell lines was also evaluated in presence of exogenous uridine (100 µM). To calculate the percentage of cell growth rate using CCK8, the following protocol was used:


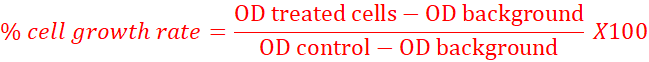


**Cell proliferation**

The proliferation index of CML CD34+ cells was evaluated by flow cytometry. Briefly, cells were incubated with 1 µM carboxyfluorescein diacetate succinimidyl ester **dye** (CFSE, Vybrant CFDA SE cell tracer kit; Molecular Probes, Invitrogen Carlsbad, CA) for 30 min at 37°C. Then they were washed with PBS and incubated in complete media for 30 minutes at 37°. After a wash, labeled CML CD34+ cells were cultured with or without Meds433 (range 1 nM-1 µM) for three consecutive days. At the end of cultures, cells were collected and the proliferation index was quantified according to the CFSE signal intensity using FCS Express software.

**Cell cycle analysis**

Cell cycle was measured by Vybrant DyeCycle orange stain (Thermo Fisher) according to the manufacturer’s instructions. CML CD34+ and CML cell lines were treated with Meds433 from 1 nM to 1 µM for 72 hours. Cells were collected, washed with Phosphate buffered saline (PBS), and incubated at 37 °C for 30 min with the dye. Samples were acquired by FACSVerse and data were analyzed with Kaluza software.

**Immunohistochemistry**

Immunohistochemical staining was performed on paraffin-embedded tumor samples through the indirect biotin-streptavidin method using a monoclonal rabbit anti-human Ki-67 antigen (#15580) (Abcam, Cambridge, UK). All slides were processed with Vectastain ABC kits (Vector Laboratories, Burlingame, CA USA) and were visualized with 3,3'-diaminobenzidine (ImmPact DAB EqvSubstrate KIT,Vector Laboratories). Slides were counterstained with Hematoxylin.

**Plasma level of Meds433 and its metabolite in treated mice**

Plasma samples of treated mice were analyzed using a high-resolution mass spectrometer (HRMS) (Q-Exactive Orbitrap, Thermo Scientific) coupled to an HPLC instrument (1200 system Agilent). Chromatographic separation was carried out on an Ascentis C18 column (150 × 2.1 mm; 2.7-μm particle size, Sigma-Aldrich, St. Louis, MO) maintained at 35 °C. The elution mixture was composed of solvent A (HCOOH 0.1% in water) and solvent B (acetonitrile). The elution gradient was from 10 to 99% of solvent B in 12 minutes; hold at 99% for 4 minutes and re-equilibration for 4 min at 10% of solvent B. The volume of injection and flow rate were 4µL and 160µL/min, respectively. The mass spectrometric analyses were performed in positive ion mode using a HESI II source and with the following conditions: heated capillary temperature 320 °C, spray voltage 3 kV (positive ions), auxiliary gas temperature 200 °C, flow rate 6, sheath gas ﬂow rate 32, sweep gas ﬂow rate 2 (arbitrary units). Accurate mass measurements were obtained with full scan mass acquisition (resolving power R = 35,000; mass range: m/z = 200-700 Da). Data were acquired and elaborated using Xcalibur Browser (Thermo Fisher Scientific, Waltham, MA, USA). For sample preparation, fifty microliters of plasma were diluted with 200μL of ethanol containing 5 ng of M489, used as internal standard. Samples were vortexed and centrifuged at 13 200 rpm for 15 min; 4μL aliquots of the supernatants were injected directly into the HPLC-HRMS system.

**Differentiation markers**

CML CD34+ cells or CML cell lines were treated with 100 nM Meds433 for 72h and stained with anti-human CD11c-APC (#130-113-584) (Miltenyi Biotec), CD318-FITC (#130-101-250) (Miltenyi Biotec), OSCAR-APC (#130-119-672) (Miltenyi Biotec), CD1c-FITC (#17-0128-42) (Thermo Fisher), CD61-FITC (#130-110-748) (Thermo Fisher), and CD41-FITC (#130-124-887) (Thermo Fisher) at room temperature for 20 min. Also, the expression of CD11c was measured in K562 cells after the treatment with 100 nM Meds433 and in the presence of exogenous uridine 100 μM. Data collection was done on FACSVerse and data were processed with Kaluza software version 2.1.

**Measurement of ROS**

The cellular ROS production of CML CD34+ cells or CML cell lines treated with 100nM Meds433 was detected by flow cytometry staining with 15 μM DCFDA for 20 min at 37°C. Then, cells were incubated with pre-warmed media for additional 5 minutes at 37°C. After a wash, fluorescence was measured by FACSVerse using Kaluza software. The ROS analysis in K562 treated cells was also performed in the presence of 100 μM uridine.

**Measurement of Mitochondrial Membrane Potential**

The mitochondria membrane potential of CML CD34+ cells was measured following the treatment with 100 nM Meds433. After staining with tetramethylrhodamine methyl ester (TMRM; Molecular Probes, Eugene, Oregon, USA), according to the manufacturer’s instructions cells were washed with PBS, and incubated at 37°C with 20 nM TMRM for 30 minutes. Cells were washed and analyzed by FACSVerse using Kaluza software. Also, the mitochondria membrane potential of K562 treated cells was measured in the presence of exogenous uridine.

**Senescence assay**

Following the treatment with 100 nM Meds433, CML CD34+ cells were stained using cellular Senescence detection kit-SPiDER-βGal (Dojindo Molecular Technologies) according to the manufacturer’s instructions. Briefly, cells were incubated with bafilomycin A1 at 37 °C for 1 h. Cells were further incubated for 30 minutes at 37 °C with then SPiDER-βGal solution. After a wash, cells were acquired using FACS Verse and analyzed with Kaluza software.

**RNA isolation and qRT-PCR for mRNA detection**

Total RNA was isolated from CML CD34+ using TRIzol® Reagent (Invitrogen Life Technologies). For mRNA detection, 250 ng of DNAse-treated RNA (RQ1, Promega) was retrotranscribed with High-Capacity cDNA Reverse Transcription Kit (Applied Biosystems). qRT-PCRs for mRNA detection wwasperformed with the SYBR® Green PCR Master Mix on cDNAs according to the manufacturer's instructions. qRT-PCRs were carried out using gene-specific primers and a 7900HT Fast Real-Time PCR System (Applied Biosystems). Quantitative normalization was performed on the expression of β-actin mRNA. The relative expression levels between samples were calculated using the comparative delta CT (threshold cycle number) method (2-∆∆CT) with a control sample or the median expression of the analyzed genes as a reference point. The following Quantitect primer assays (Qiagen) were used: Hs_PKLR_1 QT00016156, Hs_DDIT3_1 QT00082278, Hs_GPT_1 QT01001595, Hs_PLK2_1 QT00049406, Hs_CDKN1A QT00062090, HsAHSP_1 QT00215992, Hs_ACTB_1 QT000945431. The sequences of PAI-1, IL-8, TNF-α were described in (2).

**Generation of GPT1 overexpressed K562**

## K562 cell line was infected with pLenti-C-Myc-DDK-P2A-Puro lentiviral particles (Origene Technologies Inc Rockville, Maryland, USA NM_005309-# RC203756) at a multiplicity of infection (MOI) of 10. After incubation at 37°C for 20 hrs, the medium was replaced with a fresh medium. Ten days following viral infection, cells were selected by puromycin at 1 µg/ml and stable K562 cells were obtained after 7 days. The GPT1 expression level was measured and overexpression was confirmed by qRT-PCR. Puromycin-resistant cells (K562 GPT1) were used for the assays.

**Supplemental figure 1. Apoptosis and uridine rescue in CML cells treated with Meds433.** (A) demonstrates half-maximal effective concentration (EC50) that induces apoptosis in CML cells by Meds433 and/or BQ (n:3). (B) shows the percentage of apoptosis and necrotic cells in KU-812, JURL-MK1, and AR230-R (a resistant cell line to imatinib) after three days of Meds433 treatment (n:3). (C) demonstrates uridine rescue in two CML cell lines treated with Meds433 (n:3).


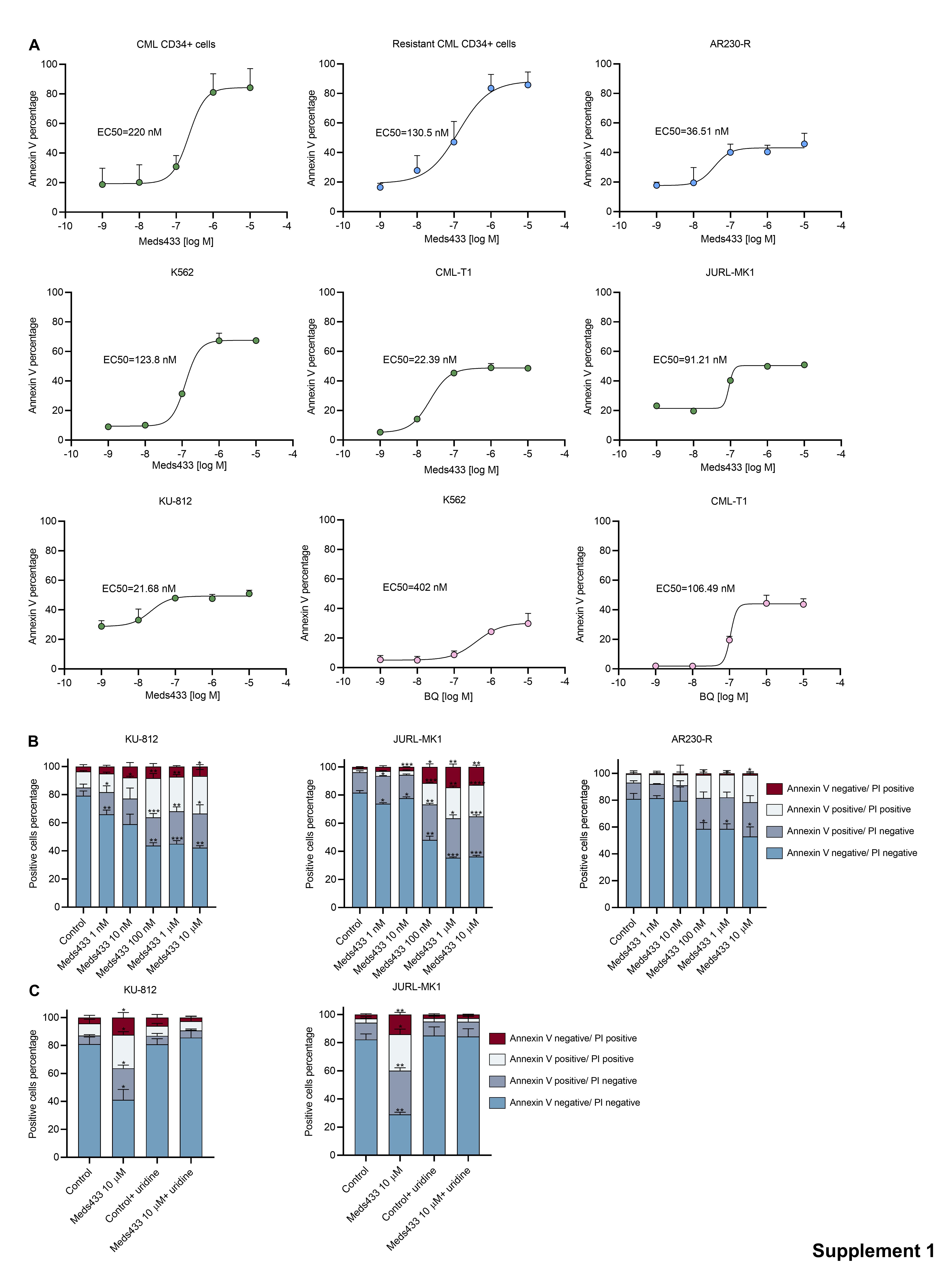


**Supplemental figure 2. Cell growth and cell cycle analysis in CML cell lines.** (A) represents cell growth analysis in KU-812 and JURL-MK1 after three days of Meds433 treatment (n:3). (B) shows cell cycle analysis of KU-812 and JURL-MK1 after Meds433 treatment (n:3).

**
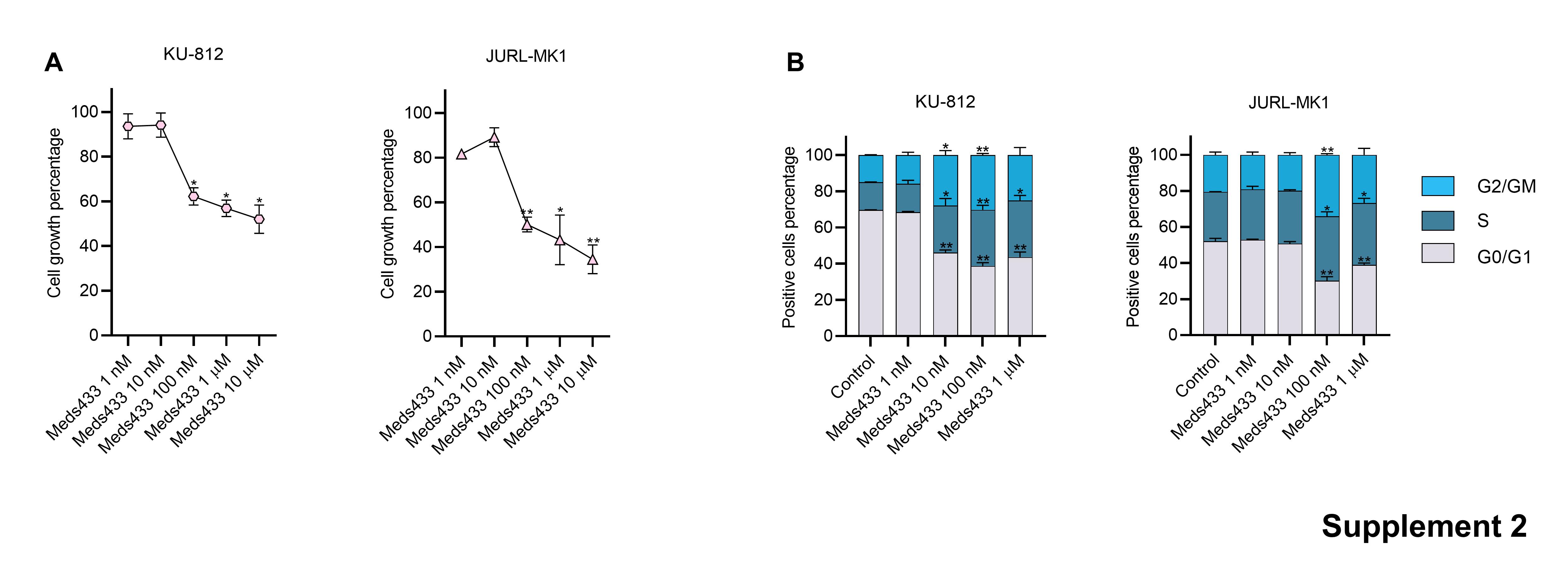
**

**Supplemental Figure 3.** H&E staining of tumors. (A) represents the images of H&E staining of tumors in the vehicle, and treated groups (10mg/kg and 20mg/kg) (20X magnification).

Supplemental figure 4. Validation of some differentially expressed genes. (A) shows some differentially expressed genes from the heatmap. Genes were selected based on the fold change. PLK2, p21 (CDKN1A), DDIT3, PKLR, and AHSP were measured in five CD34+ patient samples by qRT-PCR. Our results confirmed the trend in which genes were up or downregulated following the treatment with 100 nM Meds433.

**
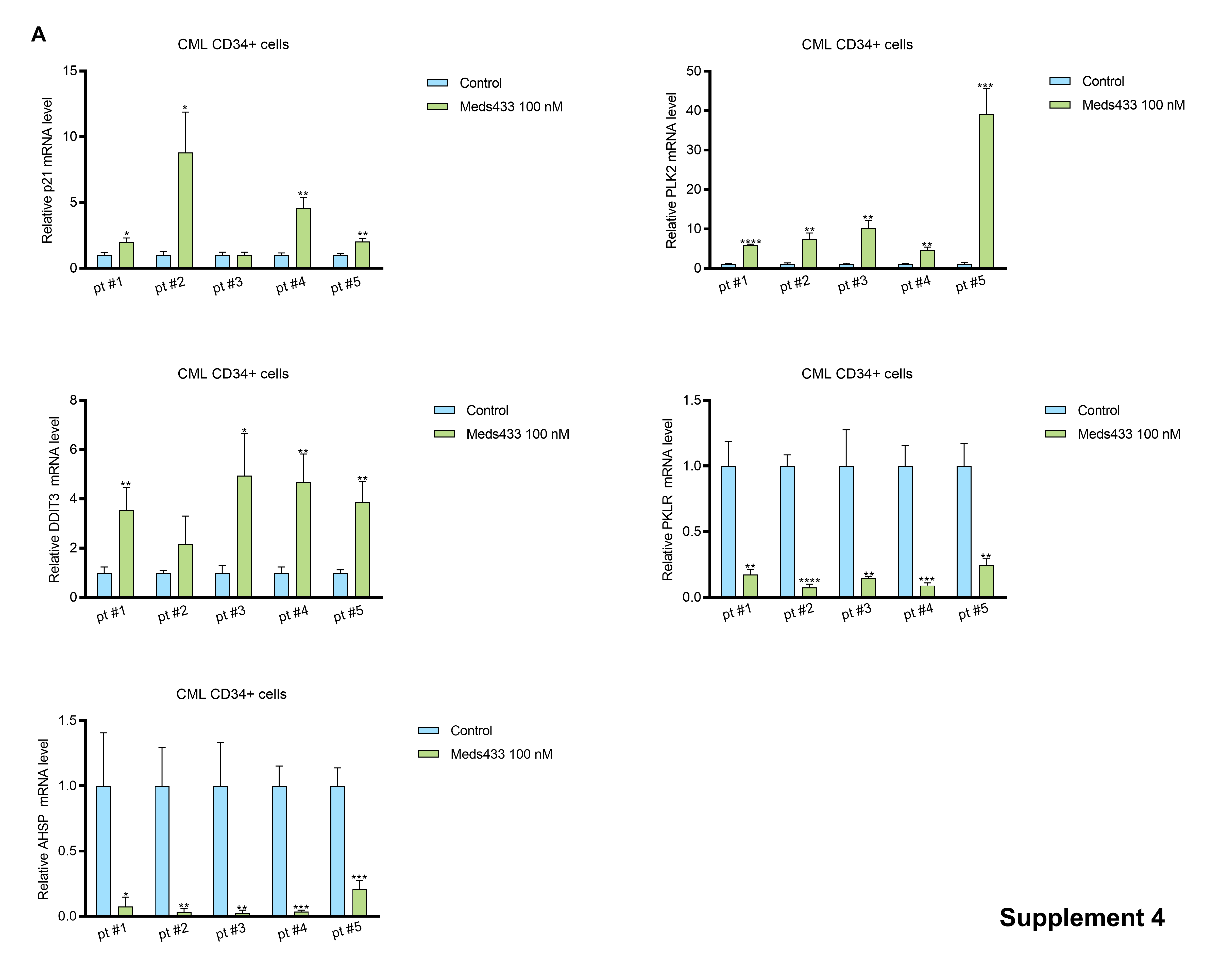
**

**Supplemental figure 5. Metabolite alterations in CML CD34+ treated cells.** (A) shows the concentration of some amino acids after the treatment of CML CD34+ cells (n:5) with 100 nM Meds433.

**
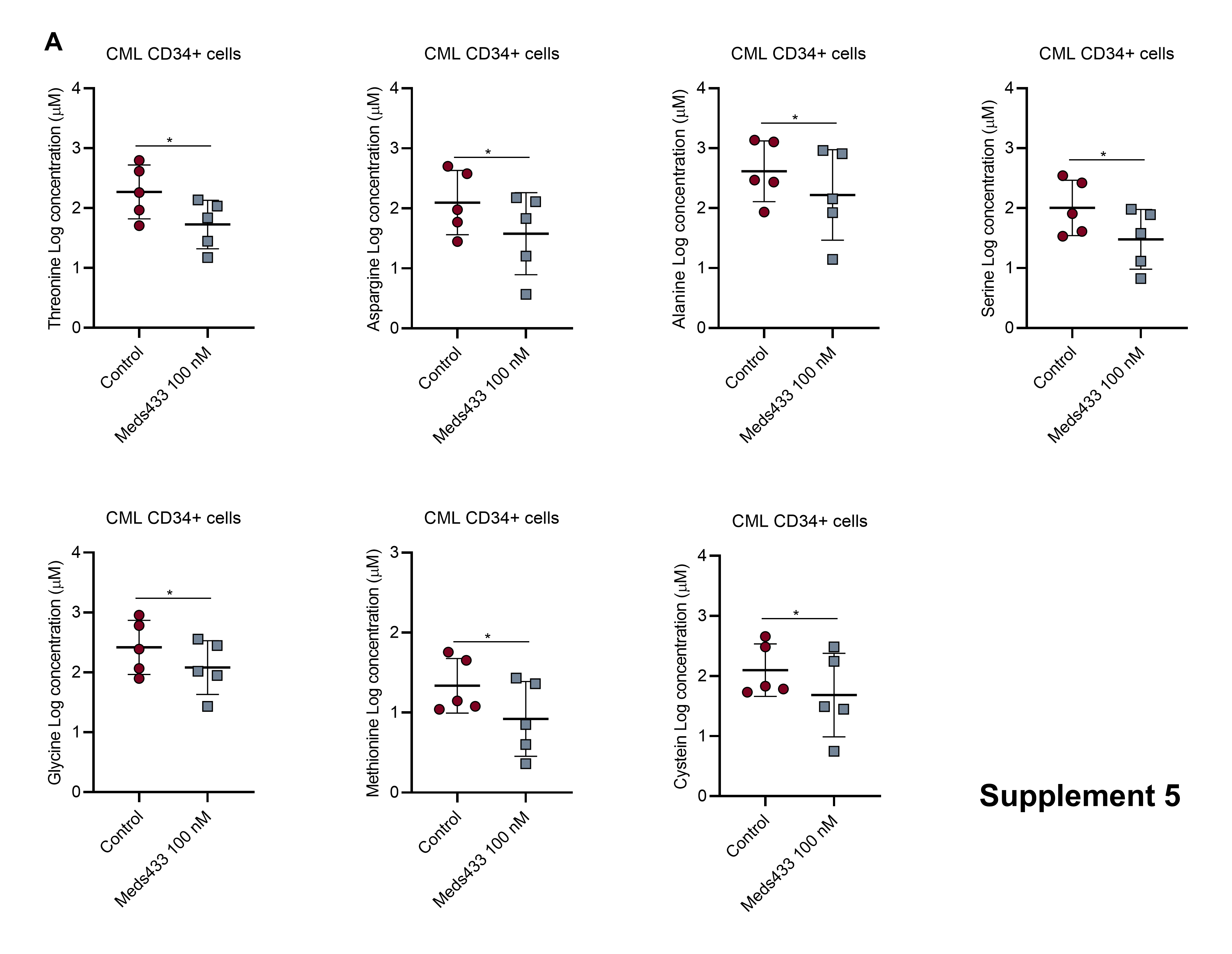
**

**Reference:**

1. Sainas S, Pippione AC, Lupino E, Giorgis M, Circosta P, Gaidano V, et al. Targeting Myeloid Differentiation Using Potent 2-Hydroxypyrazolo[1,5- a]pyridine Scaffold-Based Human Dihydroorotate Dehydrogenase Inhibitors. J Med Chem. 2018;61(14):6034-55.

2. Gulluni F, Prever L, Li H, Krafcikova P, Corrado I, Lo WT, et al. PI(3,4)P2-mediated cytokinetic abscission prevents early senescence and cataract formation. Science. 2021;374(6573):eabk0410.
