# Supplementary material for: Dihydroorotate dehydrogenase inhibition reveals metabolic vulnerability in chronic myeloid leukemia
Source: Cell Death Dis. 2022 Jun 30;13(6):576. doi: 10.1038/s41419-022-05028-9 (PMC9247109; doi:10.1038/s41419-022-05028-9)
Supplement: Supplementary file 2 — Uncropped WB [file 41419_2022_5028_MOESM2_ESM.pptx]

## Slide 1
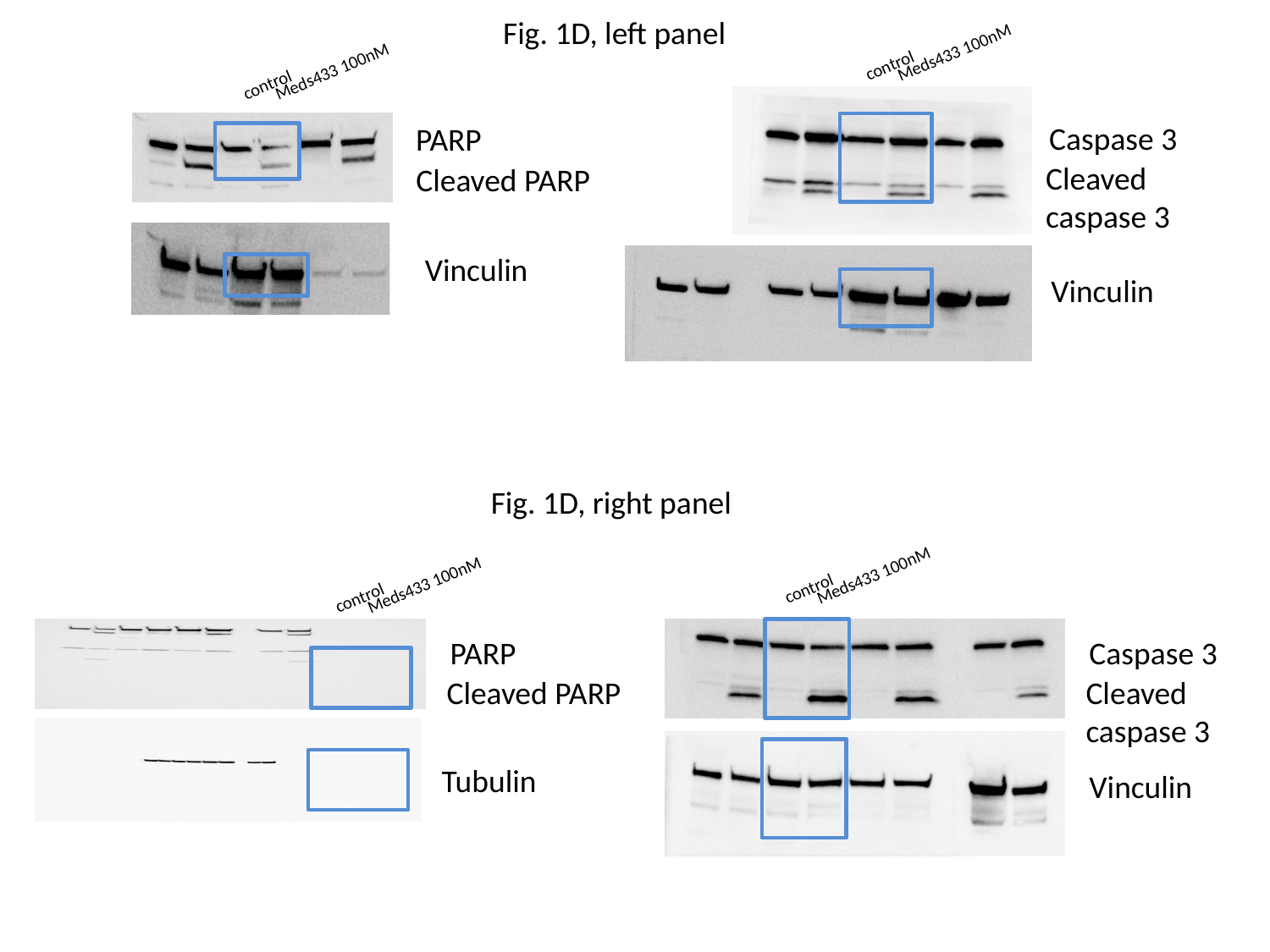

Fig. 1D, left panel
Meds433 100nM
control
Meds433 100nM
control
Caspase 3
PARP
Cleaved caspase 3
Cleaved PARP
Vinculin
Vinculin
Fig. 1D, right panel
Meds433 100nM
Meds433 100nM
control
control
Caspase 3
PARP
Cleaved caspase 3
Cleaved PARP
Tubulin
Vinculin

## Slide 2
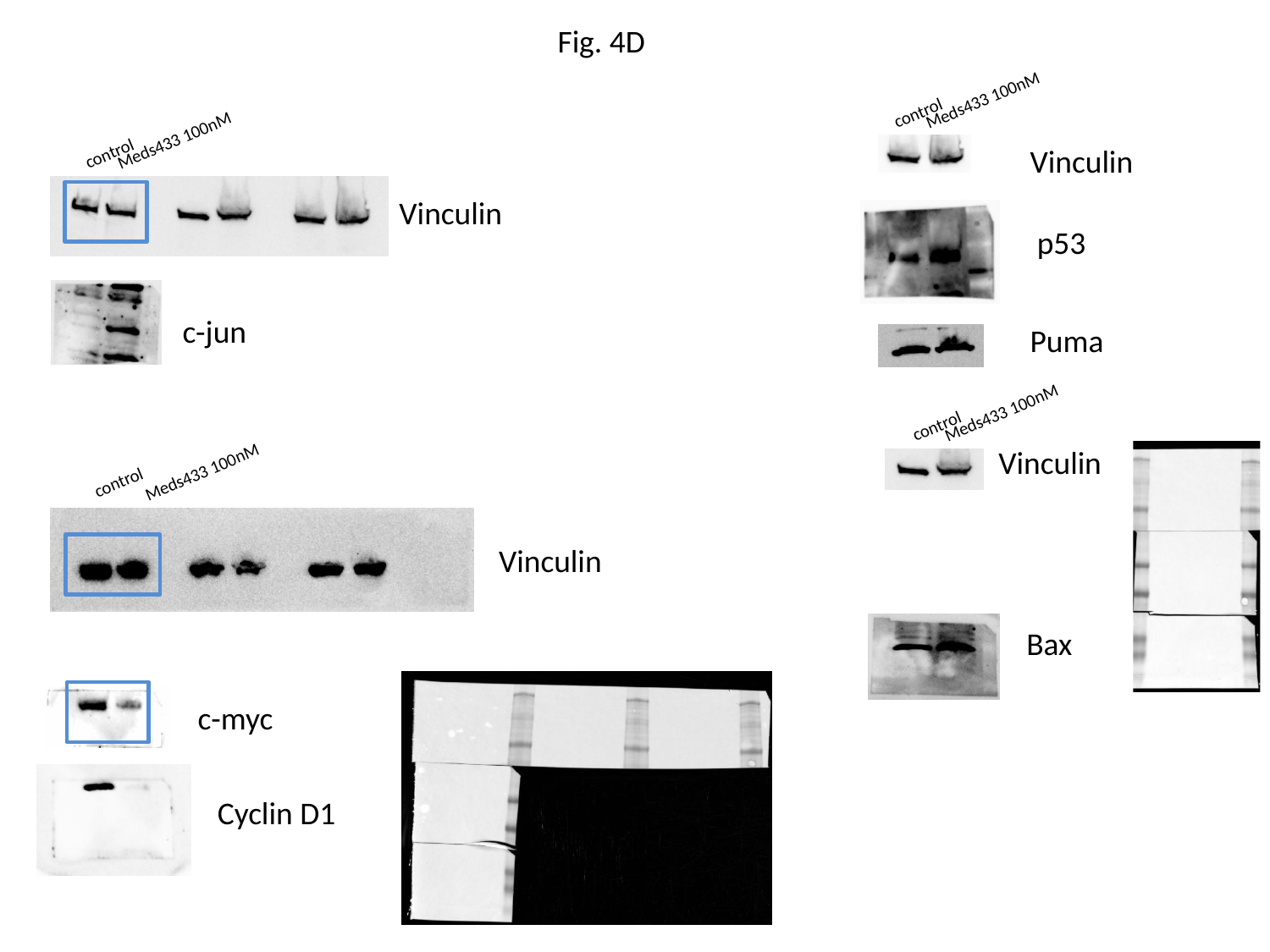

Fig. 4D
Meds433 100nM
control
Meds433 100nM
Vinculin
control
Vinculin
p53
c-jun
Puma
Meds433 100nM
control
Vinculin
Meds433 100nM
control
Vinculin
Bax
c-myc
Cyclin D1
